# The Royal General Dispensary

**Published:** 1920-11-20

**Authors:** 


### The Royal General Dispensary.

This old institution in St. Bartholomew Close celebrated the third jubilee, since its foundation in 1770, by a service at the Church of St. Bartholomew-the-Great, which the Lord Mayor and Sheriffs attended in state. The Dean of St. Paul's preached the sermon. The service was followed by a luncheon in the hall of the Butchers' Company, when the Master, Mr. William West, presided. The dispensary works in connection with St. Bartholomew's Hospital. Its task does not diminish, and it needs an extra £250 a year, for which an appeal is being made to all interested in this old charity.

